# Infections and Anti-N-Methyl-D-Aspartate Receptor Encephalitis in Children and Adolescents: A Scoping Review of Evidence Since the COVID-19 Pandemic

**DOI:** 10.3390/children13101301

**Published:** 2026-09-25

**Authors:** Olga Vampertzi, Elias Iosifidis, Androula Zelilidou, Evangelia Farmaki, Efthymia Vargiami

**Affiliations:** 1 4th Pediatric Department, Aristotle University School of Medicine, Papageorgiou General Hospital, 56429 Thessaloniki, Greece; azelili@auth.gr; 2 Infectious Diseases Unit, 3rd Department of Pediatrics, Aristotle University School of Medicine, Hippokration General Hospital, 54642 Thessaloniki, Greece; iosifidish@auth.gr; 3 Pediatric Immunology and Rheumatology Referral Center, 1st Department of Pediatrics, Aristotle University School of Medicine, 54642 Thessaloniki, Greece; farmakg@auth.gr; 4 Pediatric Neurology, Aristotle University School of Medicine, Hippokration General Hospital, 54124 Thessaloniki, Greece; evargiam@auth.gr

**Keywords:** anti-NMDAR encephalitis, autoimmune encephalitis, children, adolescents, herpes simplex virus (HSV-1), SARS-CoV-2/COVID-19, infectious triggers, neuroimmunology

## Abstract

**Highlights:**

**What are the main findings?**

- HSV-1 was the most consistently reported infectious association with anti-NMDAR encephalitis in children and adolescents, including in the post-SARS-CoV-2 era. Other infectious triggers, including SARS-CoV-2, respiratory syncytial virus, influenza B, and norovirus, were reported less frequently.
- Clinical phenotype varied with age, with younger children more frequently presenting with neurological manifestations and experiencing greater residual neurological morbidity following extensive HSV-related brain injury. Early initiation of immunotherapy was associated with more favorable neurological recovery, although the available evidence does not establish causality.

**What are the implications of the main findings?**

- Clinicians should maintain a high index of suspicion for anti-NMDAR encephalitis following HSV-1 infection, particularly in children presenting with new-onset seizures, movement disorders, or neuropsychiatric symptoms, and prioritize early neurological assessment and treatment.
- The limited and heterogeneous evidence precludes infection-specific therapeutic recommendations and highlights the need for prospective, multicenter studies with standardized diagnostic, treatment, and outcome measures to clarify the infection–autoimmunity relationship and improve long-term outcomes.

**Abstract:**

**Background/Objectives**: Anti-N-methyl-D-aspartate receptor (anti-NMDAR) encephalitis is a common autoimmune encephalitis affecting children and adolescents. Its development has been associated with preceding or concomitant infections, particularly herpes simplex virus type 1 (HSV-1). This scoping review characterized the association between infections and pediatric anti-NMDAR encephalitis, focusing on epidemiology, pathophysiology, clinical presentation, treatment, and prognosis from January 2020 to August 2025. **Methods**: A scoping review was conducted according to the Joanna Briggs Institute (JBI) framework. PubMed was systematically searched using the terms NMDAR encephalitis AND infections AND children. Of 110 identified records, 13 studies met the predefined eligibility criteria. **Results**: HSV-1 was the most consistently reported infectious association, particularly in younger children. Clinical phenotype varied with age, with neurological manifestations predominating in younger children and psychiatric, behavioral, and cognitive symptoms becoming more prominent in adolescents. HSV-associated cases, particularly following extensive HSV-related brain injury, were associated with greater residual neurological morbidity and less favorable long-term outcomes. Other infectious agents, including SARS-CoV-2, respiratory syncytial virus, influenza B, and norovirus, were reported less frequently, with limited evidence regarding their impact on disease course. Immunotherapeutic strategies were generally similar irrespective of preceding infection, although active or treatment-associated infections could influence treatment timing, sequencing, and infectious-risk considerations. Earlier immunotherapy was associated with more favorable neurological recovery. **Conclusions**: Infectious exposures, particularly HSV-1, appear to be associated with pediatric anti-NMDAR encephalitis, although causality cannot be established from the predominantly retrospective and case-based literature. Prospective, multicenter studies using standardized diagnostic and outcome measures are needed to clarify the infection–autoimmunity relationship and optimize early recognition and management.

## 1. Introduction

Anti-N-methyl-D-aspartate receptor (anti-NMDAR) encephalitis is an autoimmune encephalitis mediated by antibodies directed against the NR1 subunit of the N-methyl-D-aspartate (NMDA) receptor in the central nervous system. Among antibody-mediated autoimmune encephalitides, anti-NMDAR encephalitis is the predominant subtype in children and adolescents [1].

The disorder predominantly affects young women, particularly in paraneoplastic contexts, with a reported female-to-male ratio of approximately 4:1 and a median age at onset of 21 years. However, anti-NMDAR encephalitis has been documented across a wide age spectrum, from infancy to older adulthood [2]. Secondary anti-NMDAR encephalitis following herpes simplex virus (HSV) encephalitis is a well-established phenomenon, reported in approximately 18–30% of patients, although prevalence estimates vary depending on study design and diagnostic criteria [3,4].

Clinical presentation varies considerably with age. In adults, the disease typically manifests with prominent psychiatric symptoms, including behavioral changes, anxiety, paranoid ideation, and psychosis. In contrast, pediatric patients more commonly present with neurological features, such as seizures and movement disorders which often predominate during the early stages of the disease [5]. In 2016, Graus et al. proposed consensus diagnostic criteria for probable and definite anti-NMDAR encephalitis. A definite diagnosis requires the detection of anti-NMDAR antibodies in association with at least one of six core clinical features: psychiatric symptoms or cognitive dysfunction; speech disturbance (including mutism); seizures; movement disorders or muscle rigidity; decreased level of consciousness; or autonomic dysfunction and/or central hypoventilation [6]. Ιn 2023, Dalmau and Graus revisited the diagnostic criteria, highlighting the high frequency of misdiagnosis and the inappropriate application of the criteria, particularly in antibody-negative autoimmune encephalitis [7].

First-line treatment consists of immunotherapy, with high-dose intravenous corticosteroids forming a cornerstone of therapy due to their broad anti-inflammatory effects and ability to penetrate the blood–brain barrier [1]. Intravenous immunoglobulin (IVIG) and plasmapheresis (PLEX) are commonly employed as alternative or adjunctive first-line therapies, particularly in patients with an insufficient response to corticosteroids [8].

Increasing evidence suggests that infectious events may precede the onset of anti-NMDAR encephalitis, particularly in children and adolescents, raising the possibility that infections may act as immunological triggers in susceptible individuals [9]. This association has gained particular relevance since the onset of the COVID-19 pandemic, a period characterized by substantial changes in the epidemiology and circulation of infectious pathogens, particularly among pediatric and adolescent populations.

Accordingly, we restricted our search to studies published from January 2020 onward, encompassing the period during which the COVID-19 pandemic substantially altered the global infectious disease landscape. The review was deliberately restricted to children and adolescents (0–18 years) because pediatric anti-NMDAR encephalitis differs from adult disease in clinical phenotype, tumor association, diagnostic presentation, and developmental context; neurological manifestations such as seizures and movement disorders are particularly prominent in younger patients. Importantly, the review was not designed to evaluate only HSV-1 and SARS-CoV-2. Rather, it aimed to map all infectious agents reported in temporal association with pediatric anti-NMDAR encephalitis during the predefined period; HSV-1 and SARS-CoV-2 emerged from the retrieved literature as the most frequently discussed agents, while RSV, influenza B, norovirus, and HHV-6 were represented by sparse or isolated reports. This scoping review therefore aims to map and synthesize the available evidence on infectious exposures temporally associated with anti-NMDAR encephalitis in children and adolescents, with emphasis on epidemiology, proposed immunopathogenic mechanisms, clinical presentation, management, outcomes, and gaps in the post-2020 evidence base.

## 2. Materials and Methods

### 2.1. Study Design

This study is a scoping review conducted in accordance with the Joanna Briggs Institute (JBI) methodological framework and reported in accordance with the Preferred Reporting Items for Systematic Reviews and Meta-Analyses extension for Scoping Reviews (PRISMA-ScR) guidelines [10].

A scoping review design was selected because the objective was to map the breadth, characteristics, and gaps of a heterogeneous evidence base spanning retrospective cohorts, observational studies, case series, and case reports, rather than to estimate a pooled treatment effect. The completed PRISMA-ScR checklist is provided in the Supplementary Materials. This review was not registered in a publicly available protocol registry.

### 2.2. Objectives

The review was guided by the following predefined research questions:

1. What epidemiological data are available regarding infections in pediatric and adolescent patients with anti-NMDAR encephalitis?
2. Which pathophysiological mechanisms have been proposed to explain the association between infections and anti-NMDAR encephalitis?
3. How do infectious events influence clinical presentation and disease course in children and adolescents?
4. What is the impact of infections on therapeutic decision-making and clinical management?
5. How do infections affect prognosis and clinical outcomes in pediatric and adolescent anti-NMDAR encephalitis?

### 2.3. Search Strategy

A literature search was conducted exclusively in PubMed using the Advanced Search function. The search combined the concepts “NMDAR encephalitis,” “infections,” and “children.” The publication period was restricted to January 2020 through August 2025 and the language to English. The initial search yielded 110 records; 74 records published before January 2020 were excluded, leaving 36 records for eligibility screening. Reference lists of the included full-text articles were also manually screened to identify potentially relevant evidence not captured by the database search. PubMed was selected as the sole bibliographic database for this focused scoping review because the objective was to map recent, clinically oriented biomedical literature on infection-associated pediatric anti-NMDAR encephalitis within a narrowly defined population, concept, context, and time period. However, we acknowledge that restricting the electronic search to a single database may have resulted in the omission of relevant studies indexed exclusively elsewhere and represents an important methodological limitation of this review. The author-contribution statement identifies O.V. and A.Z. as responsible for the literature search; because the submitted manuscript did not prospectively document an independent duplicate-screening procedure or a formal discrepancy-resolution algorithm, no such procedure is claimed in the revised manuscript.

### 2.4. Eligibility Criteria

Studies were selected based on predefined inclusion and exclusion criteria, following the Population–Concept–Context (PCC) framework.

#### 2.4.1. Inclusion Criteria

- **Population:** Pediatric and adolescent patients (0–18 years) diagnosed with anti-NMDAR encephalitis.
- **Concept:** Documented association between anti-NMDAR encephalitis and infectious agents, including viral, bacterial, or other pathogens.
- **Study design:** Primary research studies, including case reports, case series, and observational studies.
- **Language:** English.
- **Publication period:** January 2020 onwards–August 2025.

#### 2.4.2. Exclusion Criteria

- Studies involving exclusively adult populations.
- Articles lacking reference to infectious factors or providing insufficient documentation of the infection–encephalitis association.
- Review articles, editorials, commentaries, or theoretical papers without original data.
- Articles published prior to 2020.

### 2.5. Study Selection and Data Synthesis

Following the initial screening, 36 records remained for title and abstract assessment. Nine articles were excluded because they were review articles without original data. Full texts of 27 articles were sought, and one article was excluded because the full text could not be retrieved. The remaining 26 articles underwent full-text assessment according to the predefined eligibility criteria; 13 were excluded and 13 studies were included in the final scoping review. The study selection process is presented in the PRISMA flow diagram (Figure 1), and the main characteristics of the included studies are summarized in Table 1. Findings were synthesized descriptively because of substantial heterogeneity in study design, populations, infectious exposures, diagnostic approaches, and outcome reporting.

Given the heterogeneity of study designs and reported outcomes, the findings were synthesized descriptively and organized according to the predefined research questions.

According to the author-contribution statement, O.V. and A.Z. conducted the literature search. As the original manuscript did not prospectively specify an independent duplicate-screening process or a formal procedure for resolving discrepancies, the revised manuscript does not state that such procedures were undertaken.

### 2.6. Methodological Considerations

The included evidence comprised heterogeneous study designs, including retrospective observational studies and individual case reports. Accordingly, the findings were not interpreted as methodologically equivalent, and conclusions derived from isolated case reports were treated as hypothesis-generating rather than confirmatory. A formal risk-of-bias or critical-appraisal tool was not applied in the original review process. This is acknowledged as a limitation, particularly because the strength of evidence supporting associations with non-HSV infectious agents is largely based on individual case reports.

## 3. Results

### 3.1. Epidemiology

Among the studies included in this scoping review, herpes simplex virus (HSV) was the most frequently reported preceding or concomitant infectious agent in pediatric patients with anti-N-methyl-D-aspartate receptor (anti-NMDAR) encephalitis.

Several cohorts highlighted the relevance of HSV infection, particularly among younger children. Marcus et al. [12] reported evidence of HSV infection in approximately one third of pediatric patients with confirmed anti-NMDAR encephalitis. Most affected individuals were infants, with five of the six patients being younger than one year of age, and a male predominance was observed. The median interval between HSV detection and subsequent identification of anti-NMDAR antibodies was approximately three months.

Comparable age and sex distributions were reported in the retrospective analyses by Dumez et al. [11] and Herrera-Mora et al. [14]. In these studies, HSV-associated cases were more frequently identified in pediatric patients than in adults and were characterized by a higher proportion of male patients. Specifically, Dumez et al. [11] identified HSV infection in 13 of 198 patients with anti-NMDAR encephalitis evaluated between 2014 and 2022. Among these patients, four (31%) were younger than four years of age, while males accounted for 54% of cases. Similarly, Herrera-Mora et al. [14] reported that, among 66 patients ranging from the neonatal period to 35 years of age, 60.6% were younger than 18 years. HSV infection was identified in 35% of pediatric patients, compared with 7.7% of adults, with a male predominance among affected patients (60%).

Other infectious agents were reported less frequently across the included studies. These included SARS-CoV-2 [16,17,18], norovirus [22], influenza virus [21], and respiratory syncytial virus (RSV) [20]. Notably, reports of SARS-CoV-2-associated cases [16,17,18], emerged during the COVID-19 pandemic and post-pandemic period, adding to the spectrum of infectious exposures temporally associated with anti-NMDAR encephalitis in children and adolescents.

### 3.2. Pathophysiology

Among the studies included in this scoping review, herpes simplex virus (HSV) was the most frequently reported infectious agent for which potential pathophysiological mechanisms underlying anti-N-methyl-D-aspartate receptor (anti-NMDAR) encephalitis were proposed [11,12,13]. Several studies proposed molecular mimicry as a potential mechanism, whereby viral epitopes may share structural similarities with neuronal antigens, potentially contributing to the initiation of an autoimmune response.

Beyond HSV, human herpesvirus 6 (HHV-6) was identified in one included study as a potential infectious trigger of anti-NMDAR encephalitis [19]. The proposed mechanisms included immune-mediated neuronal injury and subsequent autoantibody production, similar to mechanisms described following HSV infection.

Several included studies also reported temporal associations between severe acute respiratory syndrome coronavirus 2 (SARS-CoV-2) infection and anti-NMDAR encephalitis [16,17,18], Proposed mechanisms included molecular mimicry between SARS-CoV-2 epitopes and the NR1 subunit of the NMDA receptor, as well as infection-related immune activation. These mechanisms were proposed as potential pathways through which SARS-CoV-2 infection may contribute to the development of autoimmune neurological manifestations.

One included case report described the development of anti-NMDAR encephalitis in a 14-year-old female patient with an ovarian teratoma following influenza B infection [21]. In this case, peripheral inflammation associated with the viral infection was proposed to contribute to disruption of the blood–brain barrier, potentially facilitating access of circulating anti-NMDAR antibodies to the central nervous system. Binding of these antibodies to NMDA receptors, which are abundantly expressed in limbic structures, was subsequently proposed to contribute to the development of encephalitis.

Additional viral pathogens reported less frequently among the included studies included influenza B virus, respiratory syncytial virus (RSV), and norovirus [20,21,22]. The mechanisms proposed in these reports included systemic inflammation, blood–brain barrier disruption, and immune activation preceding the onset of neurological symptoms.

### 3.3. Clinical Presentation and Outcomes

Across the studies included in this scoping review, preceding or concomitant infections were reported in association with the onset and clinical manifestations of anti-N-methyl-D-aspartate receptor (anti-NMDAR) encephalitis. Herpes simplex virus (HSV) was the most commonly identified infectious agent preceding or accompanying anti-NMDAR encephalitis.

Marcus et al. [12] compared patients with anti-NMDAR encephalitis following confirmed herpes simplex encephalitis (HSE + NMDAR) with those diagnosed with primary anti-NMDAR encephalitis. HSE + NMDAR cases predominantly involved pediatric patients, particularly infants younger than one year of age. In this group, seizures were the most common initial manifestation, whereas behavioral disturbances and dyskinesias were generally absent at disease onset. In contrast, patients with primary anti-NMDAR encephalitis more frequently presented with early behavioral or psychiatric symptoms, while seizures were less commonly reported at initial presentation. During disease progression, all HSE + NMDAR patients developed seizures, and approximately half subsequently exhibited behavioral abnormalities and movement disorders, some of which persisted beyond hospital discharge.

Differences in long-term outcomes were also reported. In the retrospective study by Dumez et al. [11], patients with HSE + NMDAR demonstrated poorer recovery of neuropsychiatric function at three- and twelve-month follow-up compared with patients with primary anti-NMDAR encephalitis. Persistent seizures requiring antiepileptic treatment, memory impairment, and behavioral disturbances were frequently documented. Neuroimaging findings also differed between groups, with structural brain lesions reported in all HSE + NMDAR patients and described as more extensive than those observed in primary anti-NMDAR encephalitis.

Age also appeared to influence the clinical phenotype and disease course. One included study reported that younger age at onset was associated with distinct clinical phenotypes and disease trajectories [14]. Across pediatric cases, early manifestations were predominantly neurological and included altered level of consciousness, seizures, ataxia, dystonia, and dyskinesias.

In the context of the COVID-19 pandemic and subsequent period, three included reports described pediatric anti-NMDAR encephalitis temporally associated with SARS-CoV-2 infection [16,17,18], involving patients aged 7 years, 14 years and 23 months. The toddler and 7-year-old predominantly exhibited neurological manifestations, including seizures, abnormal limb movements or ataxia, and altered consciousness, whereas the 14-year-old also demonstrated prominent behavioral disturbance, sleep disorder, and facial dystonia. Although this pattern is broadly compatible with the recognized age-related shift from predominantly neurological manifestations in younger children toward more behavioral and psychiatric features in adolescents, the evidence consists of only three individual reports and does not permit an age-specific inference. The reports should therefore be interpreted as descriptive observations rather than evidence that SARS-CoV-2 determines a distinct age-related phenotype. Temporal association alone does not establish causality.

Other viral infections were reported less frequently among the included studies, including respiratory syncytial virus [20], influenza B virus [21] and norovirus [22]. In some cases, febrile seizures occurring in the context of neurotropic viral infections preceded the diagnosis of anti-NMDAR encephalitis [22].

### 3.4. Therapy

Across the studies included in this scoping review, treatment approaches for anti-N-methyl-D-aspartate receptor (anti-NMDAR) encephalitis were broadly comparable between patients with and without a preceding infectious trigger. In a large cohort of 198 patients, Dumez et al. [11] reported similar first-line immunotherapeutic approaches among patients with primary anti-NMDAR encephalitis and those with documented preceding infections, including herpes simplex encephalitis (HSE). High-dose intravenous corticosteroids and intravenous immunoglobulin (IVIG) were the most frequently reported first-line treatments, whereas plasmapheresis was used less frequently. Among patients with an inadequate response to first-line therapy, escalation to second-line immunotherapy, most commonly rituximab, was also reported at comparable rates between the two groups.

A difference in treatment intensity was reported in one study. Marcus et al. [12] found that patients with primary anti-NMDAR encephalitis received a greater number of immunotherapeutic interventions than those with secondary, infection-associated anti-NMDAR encephalitis.

Several studies also reported infections occurring during immunosuppressive treatment. One study described severe infections and fatal outcomes in pediatric patients who developed profound lymphopenia following rituximab therapy [23]. In individual case reports, the presence of an active infection affected the timing or sequence of immunotherapy. For example, in a seven-year-old boy with COVID-19-associated secondary anti-NMDAR encephalitis, corticosteroid treatment was postponed because of concerns regarding the ongoing active infection [16].

### 3.5. Prognosis

In the retrospective cohort study by Dumez et al. [11], which included 198 patients with confirmed anti-N-methyl-D-aspartate receptor (anti-NMDAR) encephalitis, patients with a preceding history of herpes simplex virus (HSV) encephalitis had poorer neurological outcomes than those without prior HSV infection. Thirteen patients documented HSV encephalitis and showed higher rates of persistent neurological deficits during follow-up. At one year after disease onset, 92% of patients in the HSE + NMDAR group continued to experience seizures requiring antiepileptic treatment, while 77% had persistent memory impairment. Neuroimaging demonstrated extensive MRI abnormalities, predominantly involving the thalamus and basal ganglia. More extensive HSV-related brain lesions were reported among patients who subsequently developed secondary anti-NMDAR encephalitis.

Marcus et al. [12] described three infants with concurrent HSV and anti-NMDAR encephalitis who showed minimal clinical recovery; all had extensive brain lesions during the acute HSV phase. In contrast, the only adolescent in the HSE + NMDAR group achieved complete motor recovery. Among patients with primary anti-NMDAR encephalitis, 18% had residual motor deficits or persistent seizures during long-term follow-up. Paraneoplastic tumors and secondary neurological conditions, including myelin oligodendrocyte glycoprotein (MOG) antibody-associated optic neuritis, were reported in some patients with primary anti-NMDAR encephalitis, whereas no paraneoplastic or secondary neurological conditions were documented in the HSE + NMDAR group.

Early initiation of immunotherapy was associated with more favorable neurological outcomes across the available studies. Brisca et al. [15] reported that initiation of immunotherapy within seven days of symptom onset was associated with improved neurological recovery. Similarly, Herrera-Mora et al. [14] found that earlier treatment was associated with more favorable long-term outcomes, irrespective of whether a preceding infectious trigger was documented.

## 4. Discussion

### 4.1. Epidemiology

Epidemiological evidence regarding infectious exposures and pediatric anti-NMDAR encephalitis remains limited and heterogeneous. HSV is the most consistently documented antecedent, but the present review should be interpreted as a post-2020 mapping review rather than a comprehensive historical synthesis of post-HSV autoimmune encephalitis. A 2025 systematic review and meta-analysis by Cleaver et al. [24]. searched Embase, Medline, PubMed, and Web of Science across 2007–2024 and included 78 studies comprising 225 patients with autoimmune encephalitis after HSV encephalitis, demonstrating that a much broader HSV-specific evidence base exists when wider databases, age groups, years, and autoimmune encephalitis phenotypes are considered. This difference highlights the narrower pediatric, anti-NMDAR-focused, post-2020 scope of the present review, while also underscoring the need to ensure that the search strategy is sufficiently sensitive.

Previous multicenter studies [3,4] have provided evidence of an association between HSV encephalitis and subsequent anti-NMDAR autoimmunity. Prüss et al. [3] identified anti-NMDAR antibodies in 13 of 44 patients during the acute phase of HSV encephalitis, while Armangué et al. [4] subsequently reported the development of anti-NMDAR antibodies in nearly one fifth of patients within two months following HSV encephalitis. Collectively, these findings support HSV as the most consistently recognized infectious antecedent of anti-NMDAR autoimmunity and provide a biological basis for the occurrence of secondary anti-NMDAR encephalitis following HSV encephalitis.

Beyond HSV, several other infectious agents have been reported in association with anti-NMDAR encephalitis, including cytomegalovirus, adenovirus, Epstein–Barr virus, and *Mycoplasma pneumoniae*. The latter has been reported in a substantial proportion of patients in some cohorts [25], although the strength and consistency of these associations remain less well established than those observed for HSV. These observations are particularly relevant to pediatric populations, in whom several of these infections are relatively common. More recently, SARS-CoV-2 infection has also been reported in association with anti-NMDAR encephalitis, including in children and adolescents, expanding the spectrum of infectious exposures considered in the post-COVID-19 literature [26].

Additional, albeit indirect, support for a possible infectious contribution comes from reported seasonal and geographical patterns. Pellegrino et al. [27] described a higher incidence of autoimmune encephalitis during winter and spring and in regions farther from the equator, patterns that may parallel the epidemiological distribution of respiratory infections. However, these observations do not establish a causal relationship between infectious exposure and anti-NMDAR encephalitis and should therefore be interpreted cautiously.

Overall, the available evidence supports an association between infections and anti-NMDAR encephalitis, with the strongest and most consistently documented relationship involving HSV. Other infectious agents, including SARS-CoV-2, have also been reported, particularly in the more recent literature, but their contribution to the development of anti-NMDAR encephalitis remains less clearly defined. Further pediatric studies are needed to clarify whether specific infectious exposures are associated with distinct epidemiological or clinical phenotypes.

### 4.2. Pathophysiology

The findings of this scoping review are consistent with previous international reports describing a temporal association between herpes simplex virus (HSV) infection and the subsequent development of anti-N-methyl-D-aspartate receptor (anti-NMDAR) encephalitis. Before the identification of NMDA receptor autoantibodies, the emergence of neuropsychiatric symptoms one to three months after herpes simplex encephalitis (HSE) was frequently interpreted as a relapse of the initial viral infection and was often classified as post-HSE choreoathetosis [28]. This interpretation was subsequently challenged by the landmark study of Prüss et al. [3], which demonstrated the presence of anti-NMDAR antibodies in 13 of 44 patients with confirmed HSE. Subsequent studies, including those involving pediatric populations [29] provided further evidence that persistent or recurrent neurological symptoms after clearance of HSV from the cerebrospinal fluid may reflect secondary autoimmune encephalitis rather than viral reactivation.

The mechanisms underlying this transition from viral encephalitis to secondary autoimmunity remain incompletely understood. Proposed mechanisms [2] include activation of autoreactive B and T lymphocytes and subsequent production of anti-NMDAR antibodies directed against the extracellular domain of the GluN1 subunit of the NMDA receptor. HSV-associated neuronal injury, particularly within limbic and other affected brain regions, may promote the release or exposure of neuronal antigens and thereby facilitate the development or amplification of an autoimmune response. Epitope spreading has also been proposed as a potential mechanism contributing to the emergence of secondary autoimmunity following HSV encephalitis. These mechanisms may help explain why autoimmune encephalitis can emerge after apparent virological resolution rather than during active viral replication.

The identification of SARS-CoV-2 infection among the infectious exposures reported in association with anti-NMDAR encephalitis provides a more recent context for considering infection-associated immune mechanisms. Several mechanisms have been proposed to explain a possible relationship between SARS-CoV-2 infection and autoimmune neurological manifestations, including systemic immune activation, inflammatory responses, blood–brain barrier dysfunction, and molecular mimicry [17]. However, the specific mechanisms linking SARS-CoV-2 infection to anti-NMDAR autoimmunity remain uncertain, and available evidence is largely derived from individual case reports and small series.

In pediatric patients, immune dysregulation following SARS-CoV-2 infection, including in the context of multisystem inflammatory syndrome in children (MIS-C), has been proposed as a potential contributor to autoimmune neurological manifestations. Individual pediatric cases of anti-NMDAR encephalitis occurring temporally after COVID-19 infection have been reported [18]. However, the temporal association alone does not establish causality, and alternative explanations, including coincidental infection or nonspecific post-infectious immune activation, cannot be excluded.

A similar infection-associated mechanism has been proposed following other viral infections. Phillips et al. [21], for example, suggested that peripheral inflammation following primary influenza B infection in a patient with an ovarian teratoma may have contributed to blood–brain barrier disruption and subsequent autoimmune activation. This concept is consistent with earlier observations by Irani et al. [30], who proposed that a preceding infection might act as an etiological or facilitating factor in the clinical manifestation of autoimmune encephalitis. Collectively, these observations support a model in which infection may function as an immunological precipitant in susceptible individuals, although the precise mechanisms and the factors determining progression to clinically manifest anti-NMDAR encephalitis remain to be established.

### 4.3. Clinical Presentation and Outcomes

The findings of this scoping review are consistent with international reports indicating that the clinical manifestations of anti-N-methyl-D-aspartate receptor (anti-NMDAR) encephalitis vary considerably according to age [28,30]. Desena et al. [31] described three distinct clinical phenotypes in pediatric patients, while Agarwal et al. [32] subsequently proposed a four-stage model outlining the clinical course of the disease. The findings of the present review support the relevance of both frameworks, as the included studies demonstrated age-dependent differences in clinical presentation and a clinical course characterized by an initial phase of neurological manifestations followed by behavioral, psychiatric, and cognitive symptoms in many patients.

Age-related differences in clinical phenotype have been attributed, at least in part, to developmental changes in NMDA receptor expression and function across the lifespan, potentially interacting with neurodevelopmental and age-related biological processes [33,34]. These differences may contribute to the predominance of neurological manifestations in younger children, whereas psychiatric and behavioral symptoms become increasingly prominent with advancing age.

International data suggest that a substantial proportion of patients with herpes simplex virus (HSV) encephalitis develop neuronal autoantibodies or secondary autoimmune encephalitis during follow-up, with younger children, particularly those under four years of age, reported to have earlier onset of post-HSV autoimmune manifestations and less favorable outcomes in some cohorts [4]. These observations are consistent with the findings of this review, which indicate that neurological manifestations, including seizures and altered consciousness, are particularly prominent in younger pediatric patients, whereas psychiatric and behavioral manifestations are more frequently recognized in adolescents and adults.

The potential clinical relevance of infectious exposures extends to the early manifestations of disease. Prodromal symptoms, particularly fever and headache, have been reported in a substantial proportion of patients with anti-NMDAR encephalitis, while seizures may occur early in the disease course, particularly in pediatric patients [35]. In addition, autoimmune encephalitis following HSV encephalitis may emerge after an interval of apparent virological recovery, with age-dependent neurological manifestations reported particularly in younger children [4]. Although recent viral symptoms or preceding infections may provide an important clinical context for early recognition, the available evidence is insufficient to establish febrile or seizure-related presentations as specific prodromal features of infection-associated anti-NMDAR encephalitis.

Overall, the available evidence suggests that age is a major determinant of the clinical phenotype of anti-NMDAR encephalitis. For interpretation, three clinically distinct contexts should be separated: primary anti-NMDAR encephalitis without a documented infectious antecedent; secondary anti-NMDAR encephalitis emerging after confirmed HSV encephalitis; and anti-NMDAR encephalitis reported in temporal association with other infections. The evidence is strongest for the post-HSV entity, whereas associations with other pathogens are largely based on isolated reports and should not be interpreted as proof of causation. Moreover, anti-NMDAR antibody positivity should always be interpreted together with the compatible clinical syndrome and appropriate diagnostic evaluation rather than in isolation. For non-HSV infectious agents, the available literature does not consistently demonstrate that the preceding infection independently modifies phenotype or long-term outcome.

### 4.4. Therapy

The findings of this scoping review suggest that a preceding infection has not been shown to consistently modify initial treatment decisions or subsequent therapeutic escalation in pediatric anti-N-methyl-D-aspartate receptor (anti-NMDAR) encephalitis [11]. Although some reports indicate that infection-associated cases may receive fewer or less intensive immunotherapeutic interventions than patients with primary anti-NMDAR encephalitis, this pattern was not consistently observed across studies [12].

Current international consensus recommendations support the initiation of first-line immunotherapy primarily on the basis of disease severity and clinical presentation, rather than the presence or absence of a preceding infection, and emphasize early treatment. In patients who fail to improve after approximately 2 weeks of first-line therapy, escalation to second-line immunotherapy is recommended, with rituximab generally preferred over cyclophosphamide [8]. However, the presence of an active or concurrent infection may require individualized modification of the timing, intensity, or sequencing of immunotherapy, particularly when concerns regarding ongoing infection or treatment-related immunosuppression are present [16,23].

Importantly, infection may have a dual role in the clinical course of anti-NMDAR encephalitis: it may precede or potentially contribute to the initiation of autoimmune disease, while infections may also emerge as complications of immunosuppressive treatment. Overall, the available evidence suggests that a preceding infectious trigger did not consistently result in a distinct immunotherapeutic approach; however, active or treatment-associated infections could influence the timing, intensity, or safety considerations of immunotherapy. This dual relationship highlights the need to balance timely control of neuroinflammation with careful assessment of active or treatment-associated infectious risk, particularly in pediatric patients.

### 4.5. Prognosis

The findings of this scoping review indicate that infectious exposures, particularly herpes simplex virus (HSV) infection, may be associated with long-term neurological outcomes in pediatric anti-N-methyl-D-aspartate receptor (anti-NMDAR) encephalitis. More extensive HSV-related brain lesions were associated with the subsequent development of secondary anti-NMDAR autoimmune encephalitis in the available studies, suggesting that the severity of the initial viral injury may contribute to the development of secondary autoimmune responses [11].

Across the included studies, early therapeutic intervention emerged as an important prognostic factor. Initiation of immunotherapy within the first week of symptom onset was associated with more favorable neurological recovery, irrespective of the presence of a preceding infection [14,15,36]. Taken together, the available evidence suggests that HSE-associated anti-NMDAR encephalitis may be associated with greater residual neurological morbidity, whereas earlier initiation of immunotherapy is associated with more favorable recovery. These findings support the importance of minimizing delays in immunotherapy once anti-NMDAR encephalitis is clinically suspected and appropriate diagnostic evaluation has been initiated.

Overall, international evidence indicates a clinically important association between HSV infection and poorer neurological outcomes in anti-NMDAR encephalitis. Pediatric patients, particularly infants and young children with preceding HSV encephalitis, may be at greater risk of persistent neurological deficits, potentially reflecting the severity and extent of the initial HSV-associated brain injury. Structural lesions involving the limbic system and neocortex have also been associated with poorer outcomes, including an increased risk of status epilepticus and pharmacoresistant epilepsy [37]. These findings underscore the importance of early recognition of secondary autoimmune encephalitis following HSV infection and timely initiation of immunotherapy to optimize long-term neurological outcomes.

Overall, the available evidence supports an association between infectious exposures, particularly HSV, and anti-NMDAR encephalitis, but does not establish causality. However, these findings should be interpreted cautiously because the evidence base is characterized by a predominance of retrospective studies and case reports, small pediatric sample sizes, heterogeneous diagnostic and therapeutic approaches, and limited long-term comparative outcome data. The small sample sizes and limited statistical power of the available studies do not permit firm conclusions regarding the magnitude or direction of these associations. Importantly, the absence of statistically significant findings should not be interpreted as evidence of no effect. Accordingly, the findings of this scoping review should be considered hypothesis-generating rather than confirmatory and should primarily identify potential associations and priorities for future investigation. Prospective, multicenter studies with larger pediatric populations, standardized diagnostic criteria, treatment protocols, and outcome measures are needed to better define the relationship between infection and autoimmunity and to optimize the early recognition, treatment, and long-term management of anti-NMDAR encephalitis in children and adolescents.

### 4.6. Limitations

This scoping review has several limitations that should be considered when interpreting the findings. First, the electronic literature search was restricted to PubMed and to a predefined recent publication period. Although manual reference-list screening was also performed, the use of a single bibliographic database may have resulted in omission of relevant studies indexed exclusively in other databases and should be considered an important methodological limitation. The post-2020 restriction was intentional and reflects the objective of mapping evidence emerging since the onset of the COVID-19 pandemic, rather than providing a comprehensive historical synthesis of all post-infectious anti-NMDAR encephalitis literature. Second, the available pediatric literature remains limited, with relatively few primary comparative studies and generally small sample sizes.

Third, the included studies were heterogeneous with respect to study design, patient characteristics, infectious exposures, diagnostic approaches, treatment strategies, and outcome measures. A substantial proportion of the evidence was derived from retrospective cohorts and individual case reports, which are subject to selection and reporting bias and limit generalizability. In addition, a formal design-specific quality/risk-of-bias assessment was not performed in the original scoping review. Consequently, findings from individual case reports should not be interpreted as carrying the same evidentiary weight as comparative observational studies. The predominance of observational and case-based evidence precludes firm conclusions regarding causality between specific infectious exposures and the development or clinical course of anti-NMDAR encephalitis.

Furthermore, differences in the timing of infection, diagnosis, initiation of immunotherapy, and follow-up across studies may have contributed to variability in reported outcomes. The relatively small number of pediatric patients with infection-associated disease also limits meaningful comparisons between specific infectious agents and between primary and secondary anti-NMDAR encephalitis.

Finally, the predominance of HSV-associated cases, together with the emergence of reports involving SARS-CoV-2 and other infectious agents, creates an imbalance in the evidence base and limits direct comparisons across pathogens. Publication bias is a particular concern for unusual neurological events reported after COVID-19, because temporally associated cases may be preferentially published and temporal coincidence does not establish a specific causal role for SARS-CoV-2. Outcome terminology was also inconsistent across studies; expressions such as “significant improvement,” “near-complete recovery,” or “poor recovery” were not uniformly anchored to standardized functional scales, limiting comparability. Future prospective, multicenter studies should use standardized diagnostic criteria, clearly defined infection-to-neurological-symptom and symptom-to-immunotherapy intervals, validated outcome scales, and long-term follow-up.

## 5. Conclusions

This scoping review highlights an important clinical association between infectious exposures and anti-N-methyl-D-aspartate receptor (anti-NMDAR) encephalitis in children and adolescents. Herpes simplex virus (HSV), particularly HSV-1, remains the most consistently reported infectious association, including in the post-SARS-CoV-2 era. Other pathogens, including SARS-CoV-2, respiratory syncytial virus, influenza virus, norovirus, and human herpesviruses 6 and 7, have been reported less frequently, although the available evidence remains limited and heterogeneous.

Age appears to be an important factor influencing the clinical phenotype, with younger children more frequently presenting with neurological manifestations, including seizures, movement disorders, and dyskinesias, whereas psychiatric, behavioral, and cognitive manifestations become increasingly prominent with age. HSV-associated anti-NMDAR encephalitis may be associated with greater residual neurological morbidity, particularly in younger children with extensive HSV-related brain injury. Although infection-associated immune dysregulation and subsequent development of autoantibodies targeting the GluN1 subunit of the NMDA receptor have been proposed as potential mechanisms, the biological pathways linking infection to secondary autoimmunity remain incompletely understood.

A preceding infection does not appear to mandate a distinct immunotherapeutic strategy. Early recognition and timely initiation of immunotherapy remain important for neurological recovery, while active or treatment-associated infections require individualized consideration of treatment timing, sequencing, and infectious risk. Close monitoring for infectious complications is particularly important when potent immunosuppressive therapies are administered.

Overall, the available evidence supports an association between infectious exposures, particularly HSV, and anti-NMDAR encephalitis, but does not establish causality. The predominance of retrospective studies and case reports, small pediatric sample sizes, heterogeneous diagnostic and therapeutic approaches, and limited long-term comparative outcome data substantially constrain the strength of current conclusions. These findings should therefore be considered hypothesis-generating rather than confirmatory, and the absence of statistically significant findings should not be interpreted as evidence of no effect. Prospective, multicenter studies using standardized diagnostic criteria, treatment protocols, and outcome measures are needed to better define the relationship between infection and autoimmunity and to optimize the early recognition, treatment, and long-term management of anti-NMDAR encephalitis in children and adolescents.

## Figures and Tables

**Figure 1:**
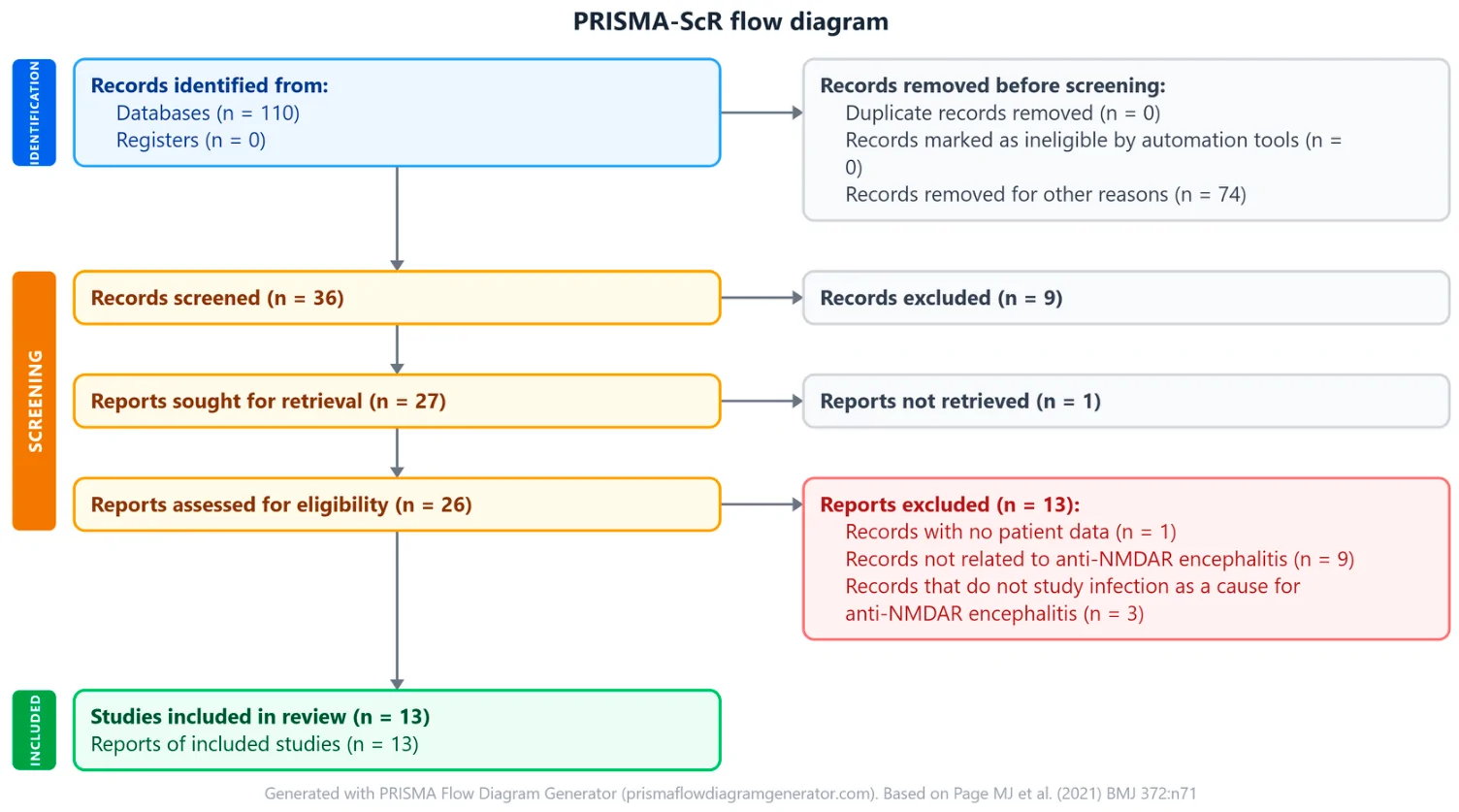
PRISMA flow diagram illustrating the study selection process, including the number of records identified through database searching, screened, assessed for eligibility, and included in the final scoping review [10].

**Table 1:** Key characteristics of studies reporting the occurrence of autoimmune anti-NMDAR encephalitis following infections in children and adolescents, including study design, population, type of infection, and main findings [11,12,13,14,15,16,17,18,19,20,21,22,23].

Authors/Year	Population	Study Type	Type of Infection	Clinical Features	Treatment/Outcome	Questions Addressed
Dumez et al., 2024 [11]	198 patients with anti-NMDAR encephalitis, 13 patients HSE + NMDAR	Retrospective	HSV (Herpes simplex encephalitis)	Behavioral changes, sleep disturbances, seizures, motor disorders, severe MRI lesions	Worse recovery in HSE + NMDAR	1, 2, 3, 5
Marcus and Ness, 2021 [12]	17 children: 6 with HSE + NMDAR 11 with anti-NMDAR encephalitis only	Retrospective comparative	HSV-1	HSE + NMDAR: seizures (100%), motor disorders (67%), behavioral symptoms (50%) Anti-NMDAR only: behavioral symptoms (100%), seizures (73%), motor disorders (73%)	HSE + NMDAR group: fewer treatments, poorer recovery–	1, 2, 3, 4, 5
Eslamiyeh et al., 2023 [13]	1 boy, 18 months	Case report	HSV	Herpes encephalitis: focal seizures, orofacial automatisms. After 30 days: chorea, ataxia, speech disorders, aphasia	Herpes encephalitis: Acyclovir for 21 days. NMDAR encephalitis: IV methylprednisolone, IVIG, aripiprazole. Significant improvement at 3 months	1, 2, 3, 4
Herrera-Mora et al., 2021 [14]	66 patients with anti-NMDAR encephalitis (16 HSV+NMDAR)	Comparative observational	HSV; other respiratory, gastrointestinal, and urinary infections	Psychiatric symptoms, seizures, motor disorders, reduced consciousness, dystonia, hypotonia	IV methylprednisolone, IVIG or plasmapheresis. Rituximab in 20 patients. Better outcome with early immunotherapy	1, 2, 3, 4, 5
Brisca et al., 2023 [15]	29 patients with autoimmune encephalitis (6 anti-NMDAR)	Comparative observational	HSV	Seizures, disturbances of consciousness and motor function	IV methylprednisolone, IVIG, acyclovir. Better recovery when treatment started <7 days from symptom onset	1, 2, 3, 4, 5
Sarigecili et al., 2021 [16]	1 boy, 7 years	Case report	SARS-CoV-2	Ataxia, altered consciousness, seizures	IVIG, plasmapheresis, followed by IV methylprednisolone	1, 2, 3, 4
Sánchez-Morales et al., 2021 [17]	14-year-old boy	Retrospective case study	SARS-CoV-2	Altered consciousness and behavior, sleep disorders, seizures, facial dystonia	IV methylprednisolone, IVIG	1, 2, 3, 4, 5
Burr et al., 2021 [18]	23-month-old girl	Case report	SARS-CoV-2	Seizures, clonic limb movements limbs, irritability, altered consciousness	IV methylprednisolone, IVIG. Full recovery 2 weeks after discharge	1, 2, 3, 4, 5
Thomas et al., 2025 [19]	12 children with HHV-6 positive CSF, including 1-year-old girl with anti-NMDAR encephalitis	Retrospective observational study	HHV-6	Febrile seizures, meningoencephalitis, ADEM, anti-NMDAR encephalitis	IV methylprednisolone, IVIG, plasmapheresis, rituximab, cyclophosphamide. Ganciclovir for 14 days (anti-NMDAR patient)	1, 2, 4, 5
Yu et al., 2023 [20]	12-year-old boy	Case report	Respiratory syncytial virus (RSV)	Ataxia, encephalopathy, relapse with dysgraphia and learning difficulties. MRI: cerebellar atrophy	IV methylprednisolone pulses	1, 3, 4
Phillips et al., 2021 [21]	14-year-old girl	Case report	Influenza B	Confusion, memory loss, motor, speech and sleep disorders, seizures, dysphagia, facial dystonia	IV methylprednisolone, IVIG, ovarian teratoma removal. Near complete recovery at 6 months	1, 2, 3, 4, 5
Liu and Xiong, 2025 [22]	15-month-old girl	Case report	Norovirus	Gait instability, reduced speech, involuntary oropharyngeal movements	IVIG, corticosteroids. Near complete neurological recovery	1, 2, 3, 4, 5
Dou et al., 2020 [23]	8 children with anti-NMDAR encephalitis	Retrospective	Secondary infections–(treatment related)	Seizures, psychiatric symptoms, cognitive impairment	Rituximab as second-line therapy. Two deaths due to severe respiratory infections	3, 4, 5

## Data Availability

No new data were created or analyzed in this study. Data sharing is not applicable to this article.

## Supplementary Materials

The following supporting information can be downloaded at https://www.mdpi.com/article/10.3390/children13101301/s1, PRISMA-ScR Checklist [10].

## Abbreviations

The following abbreviations are used in this manuscript:

anti-NMDAR encephalitis	Anti-N-methyl-D-aspartate receptor encephalitis
IVIG	Intravenous immunoglobulin
PLEX	plasmapheresis
COVID-19	Coronavirus Disease 2019
HSV	Herpes Simplex Virus
RSV	Respiratory Syncytial Virus
SARS-CoV-2	Severe Acute Respiratory Syndrome Coronavirus 2
HSE	Herpes Simplex Encephalitis
